# Medical Department of Harvard University

**Published:** 1845-09

**Authors:** 


### MEDICAL DEPARTMENT OF HARVARD UNIVERSITY.

The catalogue for 1845 exhibits this school as in a prosperous condition. It ought to be so. Boston ought to have a flourishing medical school, where general literature is cultivated with so much assiduity, and every thing else is flourishing. The number of students, last winter, was 157, upon which the circular remarks:

"The Faculty are willing to consider the increased number of pupils in their Institution, which has doubled within the last five years, as an evidence that the advantages which they offer to candidates for medical degrees are becoming better appreciated by the community. And it gives them pleasure to add, that they believe a greater number of teachers for the different medical schools of the United States, in proportion, have been taken from their graduates than from any other Institution in the country. They think that they have a right to regard this as some evidence of the success of their endeavors to give a thorough course of medical instruction."

The circular makes the following announcement:

"At a meeting of the Medical Faculty, May 29, 1841, it was

"*Voted*, That hereafter two full courses of lectures in this school be required of candidates for the degree of Doctor in Medicine. But for one of these courses a substitute may be received in a course of lectures at any other medical institution in which the number of teachers is not less than six, and in which the time occupied by lectures is not less than four months.

"If the candidate has not received a University education, he shall satisfy the Faculty of Medicine in respect to his knowledge of the Latin language and experimental philosophy. Certificates of competent persons are deemed satisfactory, without a personal examination."

We do not perceive that the prosperity of the old institutions is affected by the erection of the numerous new schools, which the last few years have witnessed. The increase of medical students seems to keep pace with the rapid growth of population, and the multiplication of schools. The Mexican war, with which we are threatened, will give employment, if it should occur, to a small army of physicians, and the large territory of Texas opens a wide field in prospect for our future graduates in medicine. Y.

